# Periodizing Exercise Medicine Prescription for Patients with Cancer: A Narrative Opinion

**DOI:** 10.1007/s40279-025-02311-5

**Published:** 2025-10-16

**Authors:** Francesco Bettariga, Robert U. Newton, Chris Bishop, Luca Maestroni, Anita Borsati, Alice Avancini, Sara Pilotto, Dennis R. Taaffe, Daniel A. Galvão, G. Gregory Haff

**Affiliations:** 1 Exercise Medicine Research Institute, Edith Cowan University, 270 Joondalup Drive, Joondalup, WA 6027 Australia; 2 School of Medical and Health Sciences, Edith Cowan University, Joondalup, WA Australia; 3 School of Human Movement and Nutrition Sciences, University of Queensland, St Lucia, QLD Australia; 4 School of Science and Technology, London Sport Institute, Middlesex University, London, UK; 5 Biomedical, Clinical and Experimental Sciences, Department of Medicine, University of Verona, Verona, Italy; 6 Department of Neurosciences, Biomedicine and Movement, University of Verona, Verona, Italy; 7 Section of Innovation Biomedicine-Oncology Area, Department of Engineering for Innovation Medicine (DIMI), University of Verona and University and Hospital Trust (AOUI) of Verona, Verona, Italy; 8 Directorate of Psychology and Sport, University of Salford, Salford, Greater Manchester UK

## Abstract

Exercise has emerged as an effective therapeutic strategy in cancer management. Long-term supervised exercise programs, including resistance training and aerobic training, can improve body composition and physical fitness, modulate circulating factors (e.g., hormones, cytokines), enhance treatment tolerance, and reduce side effects, potentially influencing cancer progression and reducing mortality risk. Despite advancements in exercise oncology, opportunities remain to refine exercise prescription through the integration of advanced methodologies such as periodization and autoregulatory programming strategies. Periodization, a systematic approach to organizing training over time, has demonstrated benefits in general fitness and clinical populations but is underexplored in cancer care. Emerging evidence suggests periodized exercise programs may enhance physical fitness (e.g., muscle strength and cardiorespiratory fitness) while mitigating treatment-related side effects (e.g., fatigue, loss of bone mineral density). Here, we examine the mechanisms underlying the benefits of periodization in exercise oncology and propose an updated framework for its implementation across the cancer continuum (e.g., neo-adjuvant, adjuvant, survivorship). Incorporating parallel or emphasis periodized models tailored to individual needs may optimize outcomes. These models of periodization and the use of autoregulatory programming strategies allow exercise intensity and volume to be modulated in response to patients’ symptoms and fatigue, ensuring safety and adherence, while aligning with the goals established by the periodized training plan. Ultimately, future research should explore complementary strategies such as nutrition and psychological periodization. By adopting a holistic and individualized approach, clinicians can improve the effectiveness of exercise interventions and ultimately enhance the quality of life for patients with cancer.

## Key Points

**Table:** 

Refining exercise prescription with advanced training methodologies, such as autoregulation and periodization, is a promising avenue for patients with cancer.
Periodized models (parallel or emphasis) enable tailored exercise prescription to optimize physical and physiological adaptations throughout the cancer continuum, yet their application in cancer setting is underexplored.
Integrating nutrition and psychological periodization alongside exercise periodization may offer a holistic approach to improve quality of life and outcomes for patients with cancer.

## Introduction

Cancer represents a significant global health challenge, contributing to high morbidity and mortality [1]. Over 50 million people are estimated to be living with cancer globally and in 2022 there were 20 million new cases and 9.7 million cancer deaths worldwide [1]. These numbers are expected to rise over the coming decades and the need for effective new cancer treatment strategies is urgent [1]. To date, lung cancer is the most diagnosed and the leading cause of cancer deaths, followed by colorectal, liver, breast, and stomach cancers [1]. Over the past few decades, researchers and clinicians have increasingly recognized exercise as both a preventative management strategy for maintaining health and a therapeutic treatment for various diseases, including cancer [2].

It is increasingly acknowledged that a structured exercise program is an effective therapeutic strategy for cancer management and serves as supportive care alongside conventional treatments [2–6]. Indeed, there is a robust body of evidence that affirms that exercise is a safe and effective neo-adjuvant, adjuvant, and rehabilitative therapy that can be employed as part of a holistic cancer treatment strategy to improve various patient outcomes (e.g., muscle strength, depression, anxiety, inflammation, immune system), including reducing mortality by ~ 40% [2–5, 7, 8]. Moreover, it is well documented that long-term supervised exercise programs can reduce body weight and fat mass while stimulating increases or preservation of lean mass in both patients with cancer undergoing active treatment and survivors, especially when coupled with dietary interventions, with the magnitude of these effects potentially influenced by the stage of cancer and treatment status [9–11]. Body composition changes induced by exercise are relevant, not only for an improvement in physical outcomes (e.g., increased skeletal muscle mass and quality, reduced fat mass), but also for the physiological effects on circulating factors (e.g., hormones, cytokines, and immune cells) that may affect disease progression [12–18]. Moreover, the changes induced by exercise can also have the potential to improve treatment tolerance and reduce toxicity driven by cancer treatments (e.g., chemotherapy, radiation therapy, immunotherapy) [19]. In addition, appropriately targeted exercise programs not only enhance muscle strength and cardiorespiratory fitness (CRF), but also improve cancer and cancer treatment-related symptoms such as fatigue, anxiety, depression, and pain, leading to enhanced quality of life [20–23]. Consequently, exercise is considered a fundamental therapeutic intervention in the management of cancer [2–5].

When considering the type of exercise undertaken, two distinct modes of exercise are commonly used: (i) resistance training (RT), and (ii) aerobic training (AT). RT is defined as exercise of very short duration generating physical exertion against a resistance (e.g., barbell, dumbbell, or weight machine) for the purpose of improving muscular strength and mass [24]. These movements are performed against an external force sufficient to limit the number of repetitions due to the accumulation of neuromuscular fatigue [24]. This stimulates neuromuscular adaptations that result in an enhanced ability to generate greater muscular force [25]. In contrast, AT encompasses continuous or intermittent exercise involving repetitive movements that can be maintained over varying durations and intensities (e.g., walking, swimming, rowing, cycling) [26]. This type of exercise targets improvements in CRF and reductions in fat mass, while also eliciting whole-body, system-level physiological adaptations that enhance metabolic health.

Although research in exercise medicine for cancer management (i.e., exercise oncology) has experienced substantial growth over the past three decades [5], there are still significant opportunities to improve both exercise prescription practices and activity monitoring protocols. A significant advancement in the sophistication of exercise prescription for cancer treatment was initially proposed in 2018 for an international trial involving men with metastatic castrate-resistant prostate cancer [27]. This trial incorporated the principle of autoregulation, which subsequently became an integral component of national guidelines for exercise oncology [4]. Indeed, given the fact that patients with cancer need to deal with symptoms and treatments (e.g., chemotherapy) as well as related side effects (e.g., fatigue), it is of utmost importance to incorporate the ability to modulate the training prescription to better align with the patient’s current capacity levels and ability to tolerate the prescribed exercise [4, 27, 28]. As such, the ability of the patient to autoregulate the exercise dosage through modulating intensity and/or volume based upon their symptoms and current state of fatigue becomes increasingly important [4, 27, 28]. In this regard, a periodized training program represents a holistic approach to organizing the training process over time, aligning with established goals [29]. Periodization provides the framework that is used to guide programming decisions about the modes and methods of training that are used to stimulate targeted adaptive responses [30, 31], which may facilitate a further improvement in the prescription of exercise medicine for patients with cancer. Indeed, a systematic approach to programming (i.e., one that considers the development of multiple physical capacities) may further enhance the health-related benefits driven by exercise (e.g., muscle strength, CRF, quality of life) or prevent the loss of health markers (e.g., bone mineral density [BMD], muscle mass), which can be affected by cancer and related treatments. By allowing programming strategies to be autoregulated, a flexible and adaptable training process can be implemented to meet the individual patient’s needs. Thus, for these reasons, different programming strategies can be aligned within the goals established by the periodized training plan, and be modified to align with the cancer continuum such as neo-adjuvant, adjuvant, rehabilitative, survivorship, and palliative care, helping patients not only adhere to exercise programs, but also to foster physical and physiological adaptations [32]. Even though different forms of periodization for general fitness and human performance have been widely examined in order to foster physical and physiological adaptions [33–35], and a few studies have also been conducted in other clinical populations (e.g., cardiovascular disease) [36, 37], considerably less is known about this concept in patients with cancer.

In the cancer setting, periodization has only recently begun to be implemented with patients [27, 28]. There is growing interest in how these strategies can be adapted to the specific challenges of cancer care [38–40]. Early foundational work has demonstrated the feasibility and potential benefits of implementing periodized training strategies with patients undergoing chemotherapy [41]. For example, Howden et al. [42] presented a model of periodization that employed modified step-loaded programming strategies with women receiving anthracycline-based chemotherapy, showing both the feasibility and patient tolerability associated with periodized training methods. Such an approach underscores the value of synchronizing training intensity and volume with treatment-related fluctuations, particularly fatigue, in order to support training adherence and maximize the benefits of training [38–40, 42]. Despite the emerging evidence supporting the use of periodized training strategies in clinical environments, the integration of these strategies into routine exercise oncology remains limited. Large-scale trials comparing different periodization models are lacking, and further research is needed to clarify the specific effects of various periodization models that employ different programming approaches (i.e., autoregulatory, velocity-based training approaches) on the physical, physiological, and psychosocial outcomes of patients with cancer. Understanding these effects will help optimize patient-centered exercise programming across the cancer continuum. Therefore, the aims of the current narrative opinion are: (1) to examine the mechanisms underlying the benefits of periodization in exercise oncology; and (2) to propose an updated approach to incorporate periodization when prescribing exercise medicine for patients with cancer.

## Periodization: What is it?

Periodization is defined as the logical integration and sequencing of training factors into mutually dependent timepoints to optimize the benefits of the training process [43]. It is considered a foundation strategy when aiming to optimize physical adaptations, which are obtained by manipulating exercise training variables such as volume, intensity, frequency, and exercise mode [44, 45]. Generally, periodization involves structuring training into cycles defined by their duration and aligned with specific targeted outcomes. In a sporting context, these cycles typically include: macrocycles (12 months), mesocycles (2–6 weeks), and microcycles (1–2 weeks) [43]. Macrocycles often account for external factors, such as holidays or seasonal variations; for instance, a reduction in training volume during breaks might serve as a transition period to promote recovery or to align with the holidays. Currently, all proposed models of periodization can be classified into three basic models of periodization: parallel, sequential, and emphasis [29]. Owing to the sequential model being more appropriate for highly trained individuals [29], this review focuses on parallel and emphasis models, which may be more applicable to clinical patients [29, 46].

From a physiological perspective, it is well known that humans should be exposed to positive stressors of different volume, intensity, and mode, to drive the performance and health benefits induced by exercise [5]. Generally, in cancer-free individuals, an inability of the body to adapt to these stressors induced by training may result in critical levels of fatigue, overreaching, and overtraining [45]. When referring to patients with cancer, the inability to accommodate positive stressors may result not only in augmented fatigue, but also in reduced physical adaptations (e.g., compromised improvements in muscle strength, CRF, and body composition), which are crucial during and after cancer [5]. The principle that governs these adaptations is based on the balance between stimulus and adaptation. Indeed, improvements occur only when the exercise training stimulus progressively increases; however, without a sufficient stimulus or if the stimulus is excessive, the individual may fail to optimally adapt, leading to a maladaptive response [45]. In light of this, the main purpose of the training plan is to gradually increase or modulate the training load while introducing variations in the training stimulus, thereby providing individuals with augmented and diverse stimuli to drive effective physical adaptations [45]. Although these principles have been extensively examined in sport [44, 45], for tactical personnel [47], and other clinical (e.g., cardiac rehabilitation) [36, 37, 48] environments, the application of specific exercise training plans in cancer remains limited [45, 49].

With this in mind, it is of utmost importance to consider not only the physical changes induced by exercise (e.g., muscle strength and CRF), but also the physiological responses including metabolic, hormonal, immune, and cell signaling alterations [50–53]. The physiological responses that occur during and after an exercise session induce fatigue and fitness after-effects, which are governed by many factors including: psychological and emotional stress [54, 55], exercise volume, intensity, frequency, and mode as well as the physical status of the person (i.e., trained or untrained as well as disease and treatment status) [56, 57]. Additionally, training frequency and sequencing are key strategies for managing fatigue [58]. Indeed, trained individuals may manage higher training frequencies and complex sequencing, whilst untrained or deconditioned individuals (e.g., patients with cancer) may require more recovery time and a structured approach to avoid fatigue [58], which may lead to a disruption in homeostasis [56]. Following the recovery phase, physiological function gradually returns to a state of homeostasis (*compensation* phase) resulting in a restoration of the individual’s performance capacity [45]. If the time between training sessions is sufficient, fatigue is dissipated and energy sources (*supercompensation* phase) are replaced, resulting in a new augmented homeostatic level [45, 59].

In this perspective, several models have been suggested to explain these responses (e.g., general adaptation syndrome and stimulus-fatigue-recovery adaption theory [60]), and among them, the fitness-fatigue paradigm describes the effect of exercise training stimuli on physical and physiological adaptations [43, 44, 61]. Originally, this paradigm assumes that each training session induces two stimuli, such as fitness (positive) and fatigue (negative) [43]. Chiu and Bradford [62] revised and demonstrated the complexity of the fitness-fatigue model, introducing multiple fitness and fatigue after-effects, which explain why individual physical qualities respond differently to variations in training. The magnitude of these stimuli is dictated by the volume, intensity, and mode of the exercise session, implying that the cumulative effects of fitness and fatigue, albeit independent of each other, are exercise specific [44, 55]. Exercise training stimuli can be built gradually over time with consistent training, and physical and physiological adaptations (i.e., fitness effects) tend to last longer than rushed efforts, emphasizing the risks of rapid unsustainable progress with the principle “too fast to ripen, too fast to rot” [43, 55, 60, 63]. However, fatigue effects occur almost immediately after training but dissipate quicker than fitness effects [43]. Ultimately, the adaptations are a function of the difference between fitness and fatigue [43]. Simply put, physical and physiological adaptations are achieved when the balance between fitness and fatigue is managed effectively [43]. In fact, the magnitude of fatigue can mask fitness responses (i.e., delayed training effects), whereas, when fatigue dissipates, adaptations can be expected (*supercompensation* phase) [43, 60, 64].

## Application of Periodization in Patients with Cancer

In cancer settings, the exercise training plan should be based on a comprehensive analysis of physical and physiological capacity, in addition to the patient’s clinical needs [4]. The factors that should be considered include: chronological and biological age, training history, health status, physical fitness level, capacity to recover, and psychological distress [45, 54, 55]. Furthermore, consideration of type and stage of cancer (e.g., early stage or advanced), cancer treatment (e.g., surgery, chemotherapy, or radiation therapy), presence of comorbidities (e.g., cardiovascular disease), and treatment-related effects (e.g., peripheral neuropathy, anxiety, depression, or fatigue), along with a patient’s needs and goals (e.g., improve muscle strength, reduce fat mass) is critical before designing an exercise training program [4]. After this, it becomes of utmost importance to examine the physical capacity of a patient with cancer, including test protocols for the assessment of muscle strength (for both upper and lower limbs), as well as CRF, which provide baseline markers of physical fitness levels [4, 45]. The field of exercise oncology is rapidly evolving towards precision exercise medicine prescription. This involves an extensive assessment of the patient’s physical, nutritional and psychological well-being, as well as their health status. By identifying the health issues that most significantly impact the patient or pose the greatest risk of morbidity and mortality, flexible programming strategies can be developed to address these most pressing concerns [4].

Periodization for sport or tactical performance is generally built around optimizing preparation for key events, for example, upcoming Olympic Games, start of the competitive season, or deployment [65–67]. For patients with cancer, the same principles apply whereby the periodized training process should be designed to maintain the highest fitness level achievable throughout the cancer continuum (i.e., neo-adjuvant, adjuvant, rehabilitative, survivorship, and palliative), modulating the training targets to prepare the patient for key events such as surgery, bone marrow transplant, and the commencement of systemic or radiation therapies [4, 38–40, 42]. For example, periodization of exercise medicine prior to surgery (i.e., neo-adjuvant therapy) should be focused on maximizing the volume and intensity of exercise to increase work capacity, which is a marker of enhanced physical fitness (i.e., muscle strength and CRF). This approach aims to reduce the decline in physical fitness associated with periods of hospitalization and treatment [68, 69]. Alternatively, for patients who do not undergo surgery, periodized exercise interventions can still be strategically applied earlier during the treatment continuum as a preparatory or ‘prehabilitation’ approach. By improving physical fitness before surgery or main treatments, patients can potentially enhance their recovery outcomes (e.g., shorter hospital stays) and reduce complications post-operatively, as well as the mortality rate [68–70]. However, it is important to acknowledge that certain treatment windows (e.g., the pre-operative period) may be relatively short, which can present challenges to fully implementing these prescription models. Therefore, feasibility and realistic expectations regarding what can be achieved within limited time frames should be considered. Furthermore, exercise medicine as an adjuvant to a course of treatment such as chemotherapy, immunotherapy, or radiation therapy may have the potential to be periodized around each dosage. For a patient undergoing chemotherapy, flexible programming strategies that allow for the autoregulation of exercise volume and intensity around individual responses to chemotherapy should aim to increase the total workload leading up to the next infusion [4]. The workload would then drop in order to accommodate the patient's reduced physical capacity for the days and weeks after the infusion, while then building again as the toxicities abate, leading up to the next infusion [4, 71–73]. The same scheme may apply for repeat radiation therapy fractions and help to manage or prevent the short- and long-term side effects of cancer treatments (e.g., nausea, loss of muscle mass, fatigue) [4, 41, 42, 74]. The application of exercise medicine as a neoadjuvant or adjuvant therapy is also relevant for improving the efficacy of systemic treatments (e.g., chemotherapy), by promoting vascularization and blood flow, which in turn can enhance drug delivery and immune function [75–77]. This may slow tumor progression, reduce treatment resistance, and ultimately contribute to improved clinical outcomes [19]. Another example is preparing patients for impact loading as a therapy method to slow or reverse bone loss due to cancer treatment (e.g., hormone therapy) in the survivorship phase. These patients must develop sufficient resilience of their musculoskeletal system to tolerate the loading of impact training. As such, they commence with traditional RT, increasing in volume and intensity, until they are deemed sufficiently strong and resilient to commence introduction of impact training exercises [78, 79]. In addition, it is worth noting that flexible programming strategies should also accommodate the possible decline in health status of patients with cancer. Indeed, it may be necessary to implement a regression, rather than a progression, in the exercise training program when patients experience treatment-related side effects or disease progression. This can involve reducing the training volume and intensity to prioritize the patient’s recovery. Specifically, owing to heightened fatigue or illness, the exercise training program should be adjusted to prevent physical and physiological exhaustion, allowing patients to regain adequate physical fitness before gradually reintroducing them to higher training loads. This approach ensures that the exercise prescription remains safe and effective throughout the cancer continuum.

### Parallel Model of Periodization in Cancer

In a parallel periodized approach, the program strategies are structured with each mesocycle simultaneously addressing multiple fitness qualities with the same degree of emphasis. For example, individuals with cancer may dedicate the same amount of time to the development of both muscle strength and CRF [29, 46]. From a practical standpoint when considering both RT and AT, this model allows for variations in training stimuli within each cycle in regard to exercise intensity, volume, and duration with both training targets receiving the same amount of focus [29, 46]. As a result, each mesocycle can be tailored to maintain or improve a broad range of physical capabilities (e.g., muscle strength and hypertrophy) and target the development of general fitness. This concurrent approach is beneficial for patients with cancer or survivors who require a broad range of general fitness adaptations to counteract disease impacts and any related side effects of the associated treatments. In turn, this allows for a more balanced progression while minimizing fatigue and supporting recovery [29, 46]. Indeed, such an approach is suitable for those patients with no training experience or physical deconditioning, to gradually expose them to an augmented training load. Training intensity for RT can be set using both objective methods, such as percentages of a specific repetition maximum (e.g., 85% of 5 repetition maximum) [80], and subjective methods, including a 0–10 scale for rating of perceived exertion or repetitions in reserve [81–83]. When programming AT, training intensity is generally tailored to maximum heart rate, heart rate reserve, maximal oxygen uptake, and rating of perceived exertion [84, 85]. These approaches allow for precise adjustments tailored to the individual’s capacity, training phase, and how they feel at that moment in time.

The strength of parallel periodization lies in the ability to target multiple training goals within each training week, although it may lack some degree of specificity because of the uniform emphasis on all components. However, further flexibility in this approach can be introduced through the autoregulation of programming factors, allowing adjustments based on individual responses. The autoregulation of training factors may also improve exercise adherence, which becomes of particular interest for patients with cancer, as exercise training can be adjusted based on individual progress and recovery [86]. However, while the complexity of this model requires careful planning, overall, parallel periodization provides a balanced approach, suitable for gradually building fitness, resilience, and health while accommodating patients’ needs.

### Emphasis Model of Periodization in Cancer

Emphasis models of periodization provide a more flexible training model that adjusts the focus of training volume and intensity within a given cycle to vary the training emphasis. For example, over the course of 2 weeks, different physical capabilities (e.g., muscle hypertrophy, strength, or CRF) may be prioritized [29, 46]. Unlike parallel periodization, which targets multiple goals simultaneously and only volume and intensity of training are adjusted within each session, the emphasis model allows for alterations in training focus so that specific physical capacities (e.g., muscle strength) are emphasized during distinct phases of the training cycle, with the primary aim being to prioritize one attribute while still targeting other factors (e.g., CRF), albeit with a decreased emphasis [29, 46]. This strategy offers a significant advantage by allowing greater focus on developing a particular physical attribute before transitioning the focus to another attribute; thus, optimizing the training process cycle for physical capacities that have been identified as requiring a specific focus or development [29, 46].

This approach can be adapted to accommodate the side effects of treatments such as chemotherapy, offering flexibility to modify the intensity, volume, or mode of training as needed. Indeed, by focusing on one primary physical attribute at a time, emphasis periodization allows for more progressive developments in that specific area. In addition, by emphasizing one key attribute while maintaining moderate work in other areas, patients can experience progress in targeted capabilities, which can enhance adherence to the exercise training program. Importantly, emphasis periodization reduces the risk of *interference* between training modes. It has been reported that AT reduces the muscle hypertrophy response to RT in patients receiving androgen deprivation therapy for prostate cancer [78]. For some patients with a high disease burden and/or treatment toxicities, the immune system and capacity to adapt to exercise training are compromised, leading to sub-optimal training adaptations. However, this approach may also require careful planning to prevent setbacks in components of fitness that are not currently prioritized, unless these components are deemed irrelevant to the patient’s specific needs. For instance, in patients with sarcopenia or cachexia, where the preservation of muscle mass is paramount, a primary focus on RT is warranted, along with dietary support to ensure an adequate protein and energy intake to maximize the maintenance of muscle mass while balancing the need for recovery. While other components such as CRF remain important, they may be considered secondary depending on the patient’s clinical condition. Additionally, this approach may not be ideal for patients with low fitness levels or limited exercise experience, as they may benefit more from a less complex training model (e.g., parallel model) to build a foundation of physical fitness tailored to their specific needs and priorities.

## Practical Applications

The field of exercise oncology has advanced significantly over the last three decades, with the adoption of exercise guidelines tailored for patients with cancer [4, 5]. To date, the majority of studies have followed general exercise guidelines to achieve health-related benefits, which include performing 8–15 repetitions for 2–3 sets at a minimum of 60% of 1 repetition maximum, twice per week for RT, and at least 30 min of moderate-intensity AT, at least three times per week [5]. This means the programming strategy is limited, potentially monotonous, and may impose a high strain on patients, making it more difficult to complete [30, 31, 87, 88]. Implementing more precise periodization approaches could further enhance the field of exercise oncology by addressing these challenges [30, 31, 87, 88]. Indeed, although adhering to exercise guidelines should augment physical fitness levels, the lack of structured programming may limit physical and physiological adaptations and basic exercise programs may constrain potential benefits [45, 60]. In addition, given the inherent individualized nature of each cancer case, with factors such as cancer type and stage, treatment history and current treatment, side effects and priority health issues, age of the patient, pre-diagnosis activity, and fitness levels to name a few, it stands to reason that each patient will require some degree of bespoke planning for their exercise program. With this in mind, we have provided three hypothetical case studies in this practical applications section to show how periodization may vary depending on some of these factors.

### Case 1

A 70-year-old male former smoker, with mild chronic obstructive pulmonary disease treated with a long-acting bronchodilator, is affected by non-small cell lung cancer of the right upper lobe, adenocarcinoma subtype (stage IIIA, T3N1M0, according to *TNM Classification of Malignant Tumours*, *8th Edition*), *EGFR* and *ALK* wild-type, programmed death-ligand 1 5%. The case is discussed within the multidisciplinary team dedicated to thoracic malignancies: the disease is considered resectable and the patient a candidate for a right upper lobectomy with mediastinal lymphadenectomy followed by adjuvant cisplatin-based chemotherapy. Preoperative pulmonary function is borderline for surgery eligibility (forced expiratory volume in 1 s < 70%). Additionally, the patient presents with low muscle mass and strength (handgrip strength < 27 kg), low CRF (maximal oxygen uptake < 15 mL/kg/min), a normal body mass index (i.e., 20 kg/m^2^), along with persistent appetite loss (weight loss of 6% in the prior 3 months) and moderate fatigue. In addition, the patient does not have any prior history of regular exercise training. The referring clinician seeks to optimize the patient’s conditions and pulmonary function to increase the chances of surgical eligibility, reduce the risk of postoperative complications, as well as reduce the burden of the symptoms (e.g., fatigue and physical deconditioning). Therefore, the patient is a candidate for an 8-week prehabilitation program.

For this case, we propose a parallel periodization model that targets the maximization of both muscle strength and mass gains as well as improvements in CRF, while also aiming to limit the reduction in physical fitness following surgery (Table 1). Block 1 is focused on introducing two moderate-intensity continuous training (MICT) sessions per week, e.g. cycling, brisk walking, or jogging, to improve CRF. In detail, the adoption of moderate-intensity AT (i.e., MICT) not only stimulates aerobic and anaerobic pathways, but also is effective in reducing excessive fat mass, especially if coupled with dietary interventions [89, 90]. In addition, we recommend incorporating two RT sessions per week: one focusing on muscle strength and the other targeting muscle mass development, to optimally prepare the patient for surgery and hospitalization. Block 2 further increases the volume and intensity of MICT and introduces high-intensity interval training (HIIT) to further enhance CRF levels (e.g., employing high and low intensity bouts of stationary cycling), and facilitate fat mass loss. Frequency is also increased to avoid more than 2 consecutive days with no planned AT to facilitate the recovery of CRF after surgery. Similarly, RT sessions increase intensity and vary volume, targeting both muscle strength and mass gains, with one session emphasizing hypertrophy and the other focusing on maximal strength.

**Table 1:** Practical applications for Case 1

Block (weeks)	Mode	Intensity, objective	Intensity, subjective	Volume	Sessions per week
Block 1 (1–4)	MICT	70% HRmax	RPE ≥ 6	≥ 30 min	2
	RT (hypertrophy)	40–60% 1RM	RIR 4–5	12–15 reps for 2–3 sets	1
	RT (strength)	60–80% 1RM	RIR 2–3	6–8 reps for 3 sets	1
Block 2 (5–8)	MICT	75% HRmax	RPE ≥ 7	≥ 40 min	2
	HIIT	80–95% HRmax	RPE ≥ 7–9	60 s high intensity, 120 s low intensity for 5 rounds for 3 sets	1
	RT (hypertrophy)	50–70% 1RM	RIR 3–4	10–12 reps for 3–4 sets	1
	RT (strength)	70–90% 1RM	RIR 1–2	6–8 reps for 4 sets	1

*d/w* days per week, *HIIT* high-intensity interval training, *HRmax* maximum heart rate, *MICT* moderate-intensity continuous training, *min* minutes, *reps* repetitions, *RIR* repetition in reserve, *RM* repetition maximum, *RPE* rating of perceived exertion, *RT* resistance training, *s* seconds

The variability of this approach ensures that both CRF and muscle strength increase as the program progresses. Indeed, for CRF, the combination of MICT and HIIT targets both aerobic and anaerobic capacities, whilst for RT both muscle strength and muscle mass growth are addressed. In addition, managing malnutrition is of utmost importance [91]. In this light, tailored nutritional counseling aimed to optimize caloric and protein intakes should be coupled with the exercise training program to favorably shape body composition. Taken together, this approach aims to optimally prepare the patient for surgery and hospitalization, reducing the burden of post-treatment symptoms such as fatigue and physical deconditioning, and minimizing post-operative complications by improving overall physical fitness and body composition, enhancing immune function, and reducing systemic inflammation.

### Case 2

A 62-year-old man, without relevant comorbidities, with stage IIIB colorectal cancer (T2N2aM0, according to the *TNM Classification of Malignant Tumours*, *8th Edition*), successfully completed surgery (right hemicolectomy with lymphadenectomy) 6 weeks previously and will undergo eight cycles of adjuvant chemotherapy with capecitabine and oxaliplatin, administered every 3 weeks (XELOX regimen). The patient presents low physical fitness levels, including reduced muscle strength (handgrip strength < 30 kg) and low CRF (maximal oxygen uptake < 18 mL/kg/min), normal body mass index (i.e., 24 kg/m^2^), cancer-related fatigue, and no prior history of exercise training. The goal is to facilitate the patient to cope with activities of daily living (e.g., stair climbing, walking) during the chemotherapy treatment, minimize side effects (e.g. fatigue and gastrointestinal), and potentially enhance chemotherapy effectiveness.

For this case, following cardiac clearance to proceed with exercise (taking into consideration the cardiotoxicity of 5-fluorouracil), we propose an emphasis periodization model based on treatment infusion to accommodate the related side effects induced by chemotherapy while, at the same time, addressing the low physical fitness levels (Table 2). Evaluation of a patient’s symptoms is particularly important the day following chemotherapy infusion because of expected increased fatigue and gastrointestinal disturbances, managed with supportive therapies such as dexamethasone and/or antiemetics. We suggest different weeks of training, based on chemotherapy delivery. In Block 1, during the non-chemotherapy infusion week, the primary focus is to accumulate a higher volume of AT, using both MICT and HIIT to improve CRF, as well as RT for muscle strength 3 days per week. In detail, we focus on AT, which may help drug delivery to the tumor site [92, 93], while RT can preserve or enhance anti-cancer signaling and innate immunity (e.g., myokines, T cells, and natural killer cells) driven by skeletal muscle mass and activation [94, 95]. Subsequently, we propose concurrent training twice per week to be performed immediately before or after the chemotherapy infusion (depending on side effects such as cardiotoxicity), with low volume and intensity, accommodating lower physical work capacity, while avoiding being sedentary and associated detraining. In detail, MICT and HIIT are coupled with cluster set RT [49], aiming to maintain physical fitness levels through the therapy period. Cluster set training is of particular interest as it incorporates short intra-set rest periods (15–45 s), which help minimize fatigue and promote better exercise adherence in patients with physical deconditioning or during cancer treatments [49]. Similarly, in Block 2, volume and frequency of AT, especially HIIT, are increased in the no chemotherapy infusion week, not only to further enhance CRF but also to further prevent physical deconditioning and worsening of symptoms associated with the cancer treatment. In line with this, during the treatment week, the exercise training program is again reduced in volume and intensity for both AT and RT to facilitate recovery from the chemotherapy infusion. It is worth noting that the principle of autoregulation should be employed to accommodate the side effects induced by chemotherapy, meaning that progression or regression of exercise prescription is tailored to a patient’s response to cancer treatment.

**Table 2:** Practical applications for Case 2

Block (weeks)	Treatment phase	Mode	Intensity, objective	Intensity, subjective	Volume	Sessions per week
Block 1 (1–6)	No chemotherapy	MICT	70% HRmax	RPE ≥ 5	≥ 20 min	1
		HIIT	70–80% HRmax	RPE ≥ 7	30 s high intensity, 90 s low intensity for 5 rounds for 2 sets	1
		RT	40–60% 1RM	RIR 4–5	10 reps for 2–3 sets	1
	Chemotherapy	MICT + cluster set RT (concurrent)	70–80% HRmax 50–70% 1RM	RPE ≥ 5–6 RIR 3–4	≥ 15 min 2 + 2 + 2 + 2 reps with 20 s intra-set rest for 1–2 sets	1
		HIIT + cluster set RT (concurrent)	70–80% HRmax 50–70% 1RM	RPE ≥ 5–6 RIR 3–4	30 s high intensity, 90 s low intensity for 7–8 rounds 2 + 2 + 2 + 2 reps with 20 s intra-set rest for 1–2 sets	1
Block 2 (6–12)	No chemotherapy	MICT	75% HRmax	RPE ≥ 6	≥ 25 min	1
		HIIT	70–80% HRmax	RPE ≥ 7	30 s high intensity, 90 s low intensity for 5 rounds for 3 sets	2
		RT	50–70% 1RM	RIR 3–4	12 reps for 3 sets	1
	Chemotherapy	MICT + cluster set RT (concurrent)	60–70% HRmax 40–60% 1RM	RPE ≥ 4–5 RIR 4–5	≥ 10 min 2 + 2 + 2 + 2 reps with 30 s intra-set rest for 1 set	1
		HIIT + cluster set RT (concurrent)	60–70% HRmax 40–60% 1RM	RPE ≥ 4–5 RIR 4–5	30 s high intensity, 90 s low intensity for 5 rounds 2 + 2 + 2 + 2 reps with 30 s intra-set rest for 1 set	1

*d/w* days per week, *HIIT* high-intensity interval training, *HRmax* maximum heart rate, *MICT* moderate-intensity continuous training, *min* minutes, *reps* repetitions, *RIR* repetition in reserve, *RM* repetition maximum, *RPE* rating of perceived exertion, *RT* resistance training, *s* seconds

Our exercise training program highlights the importance of adapting exercise prescription to align with treatment cycles. Importantly, daily fluctuations in symptoms can be expected in patients undergoing chemotherapy and appropriate adjustments should be considered to optimize adherence and adaptations. This strategy not only aims to enhance physical fitness and body composition but also to accommodate treatment-related side effects (e.g., fatigue) and recovery after treatment.

### Case 3

A 39-year-old woman with stage IIA (T2N0M0, according to the *TNM Classification of Malignant Tumours*, *8th Edition*) hormone receptor-positive, HER2-negative breast cancer, has completed breast-conserving surgery (quadrantectomy of the upper outer quadrant of the left breast and sentinel lymph node biopsy) 2 years before, followed by adjuvant radiotherapy (50 Gy in 25 fractions over 5 weeks) and adjuvant chemotherapy (doxorubicin and cyclophosphamide every 3 weeks for 4 cycles, then weekly paclitaxel for 12 cycles). Given her premenopausal status, the patient is currently undergoing hormone therapy with tamoxifen (and ovarian suppression), started 18 months ago. This patient is recreationally active (i.e., walking 4–5 days per week), with a prior history of competitive exercise training (i.e., former professional swimmer). The patient presents with osteopenia with low BMD (i.e., hip T-score of − 1.9), low muscle mass, peripheral neuropathy, and normal body mass index (i.e., 22 kg/m^2^). The primary exercise goal for this patient is to increase muscle mass and BMD lost after cancer treatments and, secondly, further improve CRF to be able to perform recreational running (e.g., 5-km races).

In this case, we propose an emphasis periodization model to address the needs of Case 3 (Table 3). Block 1 serves as the foundation to build muscle mass and strength while improving CRF. Three weekly RT sessions are included, with two focusing on strength-endurance (split into lower and upper body sessions) and the other on hypertrophy (lower body), ensuring muscle growth. In addition, RT also promotes bone remodeling and increased bone density by creating mechanical stress through muscle contraction and weight-bearing exercises [96]. For CRF, MICT and HIIT are utilized to gradually improve CRF. Block 2 introduces more progression, with an increase in both intensity and volume. In the four RT sessions (split by lower and upper body), the focus continues to shift toward greater strength gains, with this block aiming to further boost muscle strength and hypertrophy. Moreover, the addition of moderate-intensity impact training (i.e., complex training [97, 98]), which involves exercises that generate mechanical forces on bones through activities such as heel drops, box jumps, and skipping, further stimulates accretion of BMD [78, 79, 99, 100]. On the aerobic side, MICT is extended and HIIT increases slightly in volume, helping to advance CRF improvements. Last, Block 3 represents the peak phase of the emphasis periodized model. The goal here is to maximize strength and power for RT, targeting both strength and power output. In addition, by building up physical capacity through RT, this allows moderate-to-high intensity impact training to be employed (e.g., jumping, hopping, and bounding) to further promote bone remodeling and bone accretion. For aerobic conditioning, MICT is further extended, while HIIT also increases in intensity, pushing the patient closer to her goal.

**Table 3:** Practical applications for Case 3

Block (weeks)	Mode	Intensity, objective	Intensity, subjective	Volume	Sessions per week
Block 1 (1–5)	MICT	60% HRmax	RPE ≥ 6	≥ 30 min	1
	HIIT	60–80% HRmax	RPE ≥ 6–8	30 s high intensity, 60 s low intensity for 8 rounds for 2 sets	1
	RT (strength-endurance)	30–50% 1RM	RIR 5–6	15–18 reps for 3 sets	2 (split into lower and upper body)
	RT (hypertrophy)	50–70% 1RM	RIR 3–4	8–10 reps for 3 sets	1 (lower body)
Block 2 (6–10)	MICT	70% HRmax	RPE ≥ 7	≥ 45 min	1
	HIIT	60–80% HRmax	RPE ≥ 6–8	30 s high intensity, 60 s low intensity for 8 rounds for 3 sets	1
	RT + impact training (complex)	50–70% 1RM	RIR 3–4	10–12 reps for 4 sets + 10 jumps (e.g., box jumps, hops) for 3 sets	2 (split into lower and upper body)
	RT + impact training (complex)	≥ 80% 1RM	RIR 1–2	6–8 reps for 4 sets + 10 jumps (e.g., box jumps, hops) for 3 sets	2 (split into lower and upper body)
Block 3 (11–15)	MICT	80% HRmax	RPE ≥ 8	≥ 60 min	1
	HIIT	70–90% HRmax	RPE ≥ 7–9	30 s high intensity, 30 s low intensity for 8 rounds for 3 sets	1
	RT + impact training (complex)	60–80% 1RM	RIR 2–3	8–10 reps for 3 sets + 15 jumps (e.g., depth jumps, hurdle jumps) for 3 sets	1 (lower body)
	RT + impact training (complex)	≥ 85% 1RM	RIR 1–2	4–6 reps for 5 sets + 15 jumps (e.g., depth jumps, hurdle jumps) for 5 sets	3 (split into lower and upper body)

*d/w* days per week, *HIIT* high-intensity interval training, *HRmax* maximum heart rate, *MICT* moderate-intensity continuous training, *min* minutes, *reps* repetitions, *RIR* repetition in reserve, *RM* repetition maximum, *RPE* rating of perceived exertion, *RT* resistance training, *s* seconds

In conclusion, the proposed approach aims to effectively enhance muscle mass, BMD, and CRF through tailored RT, impact training and AT. This emphasis periodized model allows for improvements in the outcomes of interest without creating any interference between RT and AT.

## Conclusions

While the field of exercise oncology has dramatically advanced in recent decades, there is still potential to refine exercise prescriptions for patients with cancer through the incorporation of more advanced training methodologies, including autoregulation and periodization. Indeed, the adoption of parallel or emphasis periodized models can be employed to foster physical and physiological benefits induced by exercise or to prevent physical deconditioning associated with cancer treatments (e.g., surgery, chemotherapy). Both models allow for tailored adjustments based on individual needs, capacities, and responses, ensuring safety and efficacy. However, the application of these periodization strategies in cancer populations is underexplored, highlighting the need for further research to determine their full potential. Additionally, other considerations such as nutrition periodization, which involves the manipulation of macronutrients to align with training cycles, and psychological periodization, incorporating practices such as mindfulness, should be explored to complement the systematic approach of periodization. By integrating these models into clinical practice, clinicians and practitioners can develop individualized and effective exercise programs to improve the quality of life and overall outcomes for patients with cancer.

## Declarations

### Availability of data and material

The data that support the findings of this study are available from the corresponding author upon reasonable request.
